# Port-wine stains: molecular drivers, translational models, and emerging theragnostic directions

**DOI:** 10.1007/s10103-026-04957-7

**Published:** 2026-07-22

**Authors:** Jing Zhou, Lu Hua, Liqiang Gan, Yan wang

**Affiliations:** 1 Department of Dermatology and Medical Aesthetics, Chongqing Health Center for Women and Children, Chongqing, China; 2 NHC Key Laboratory of Birth Defects and Reproductive Health, Chongqing, China; 3 Nursing Department, Chongqing Health Center for Women and Children, Chongqing, China; 4 Thoracic Surgery Department, University-Town Hospital of Chongqing Medical University, Chongqing, China

**Keywords:** Port-wine stain, Capillary malformation, GNAQ, Pulsed-dye laser, Photodynamic therapy

## Abstract

Port-wine stains (PWS), also termed capillary malformations, are congenital, progressive microvascular anomalies that can cause lifelong cosmetic and psychosocial burden and may signal syndromic involvement such as Sturge–Weber syndrome (SWS). We performed a focused evidence synthesis integrating mechanistic literature on somatic mosaicism and vascular remodeling with clinical evidence from randomized trials, observational cohorts, and quantitative-imaging studies. Key endpoints and theragnostic concepts were summarized to inform trial design and individualized treatment strategies. Somatic activating variants in GNAQ and related signaling networks drive endothelial dysfunction, aberrant vessel maturation, and lesion progression. Pulsed-dye laser(PDL) remains the global first-line modality, but response is heterogeneous and often incomplete, particularly in thick or nodular lesions. Hematoporphyrin monomethyl ether (hemoporfin)-mediated photodynamic therapy(PDT) shows high efficacy for selected phenotypes but carries distinct adverse-event profiles. Emerging theragnostics—including dynamic optical coherence tomography(D-OCT) and computational reconstruction—enable noninvasive quantification of vessel depth and caliber, supporting phenotype-guided parameter selection and objective response assessment. A mechanistically anchored, phenotype-informed framework that integrates molecular drivers with quantitative vascular imaging can accelerate precision strategies for PWS, improve endpoint rigor, and guide next-generation combination and targeted therapies.

## Introduction

**Fig. 1:**
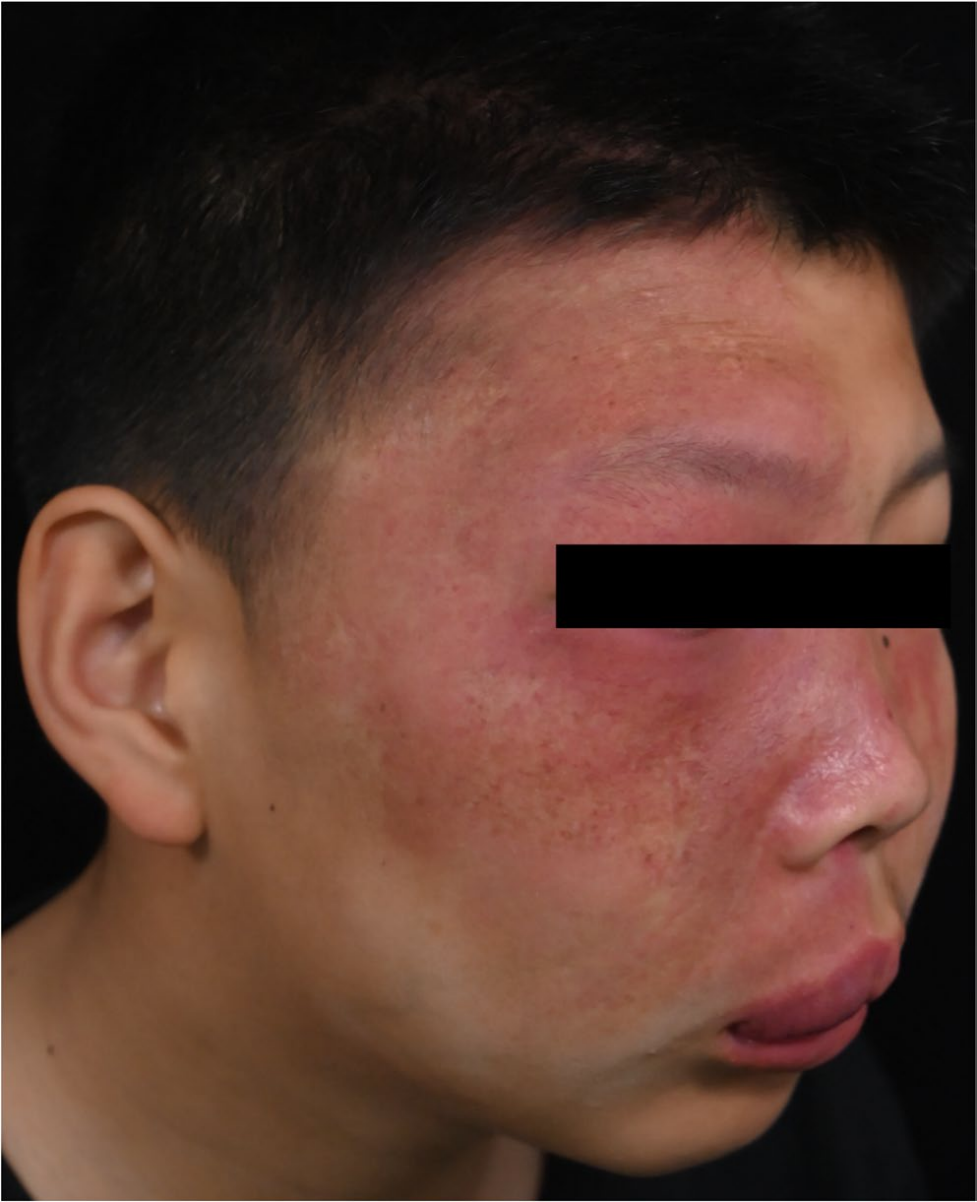
PWS, male, 12 years old, right side of the face

PWS are congenital capillary malformations characterized by ectatic post-capillary venules and progressive remodeling of the superficial dermal microvasculature [1]. The lesions typically present at birth as flat pink patches; however, with advancing age, the majority progressively darken in color and may develop vascular wall thickening, soft-tissue hypertrophy, and even nodular changes, reflecting ongoing dilation and structural reorganization of the lesional vascular network (Fig. 1) [2]. When located on exposed areas such as the face, these lesions can exert a significant negative impact on patients’ social functioning and psychological well-being [3].

Based on clinical features such as lesion color, texture, and degree of hypertrophy, PWS can be classified into red-type, purple-red-type, hypertrophic-type, and nodular-type. Histological analysis by Liu et al. demonstrated that each subtype corresponds to distinct vascular parameters, as summarized in Table 1 [4].

**Table 1:**
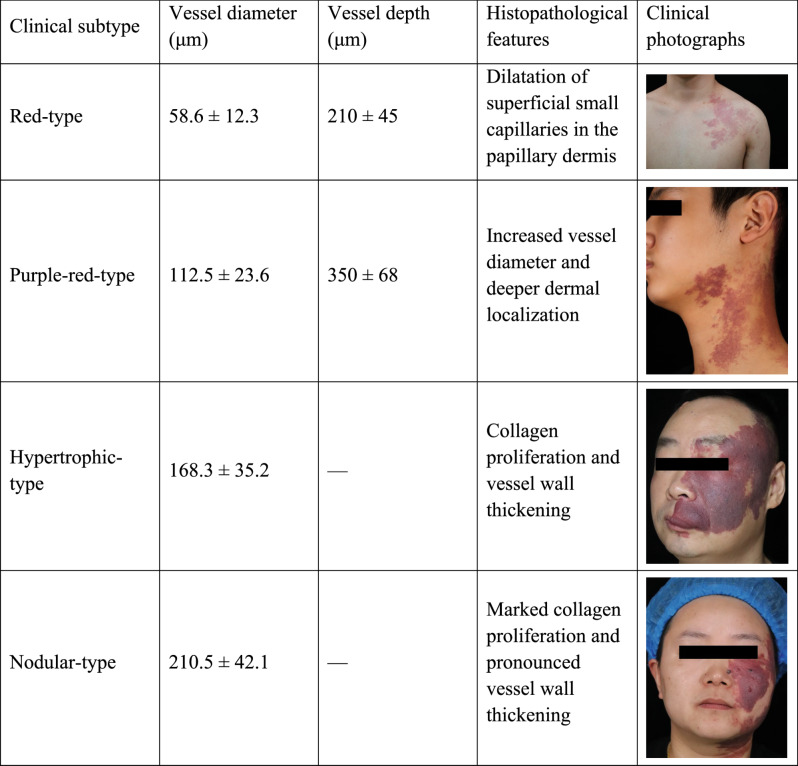
Clinical subtypes of PWS and corresponding histopathological vascular parameters

Epidemiologically, PWS affect approximately 0.3% to 0.5% of newborns and most commonly involve the face and neck [5, 6]. The visibility and chronicity of facial lesions impose a substantial psychosocial burden on patients and families across developmental stages, and late-stage hypertrophy can become functionally consequential because of bleeding, secondary infection, and distortion of local anatomy. These clinical realities drive emphasis on early diagnosis, standardized phenotyping, and timely intervention aimed at improving clearance trajectories and limiting progressive remodeling. Modern understanding of PWS has shifted from purely descriptive histology toward a molecularly grounded framework. Histopathologic studies consistently demonstrate dilated superficial dermal venules with variable perivascular and stromal remodeling, while mechanistic syntheses highlight contributions from endothelial signaling dysregulation, altered vascular tone control, and microenvironmental changes that collectively support lesion persistence and progression.

GNAQ gain-of-function mutations play a central role in the pathogenesis of PWS. Shirley et al. were the first to identify the GNAQ p.R183Q mutation, which leads to constitutive activation of the Gαq protein. This mutation is detected in 92% of isolated PWS cases and in 88% of PWS cases associated with SWS [7]. Subsequently, Galeffi et al. identified a novel mutation, GNAQ p.Q209R. Both mutations are activating mutations with similar downstream signaling effects, expanding the mutational spectrum of capillary malformation (CM)-associated mutations beyond the single R183Q hotspot [8]. Subsequent work localized mutant alleles primarily to lesional blood vessels and showed enrichment within endothelial cells, establishing a plausible genotype-to-phenotype link and an entry point for pathway-directed intervention concepts. At a functional level, endothelial GNAQ activation engages downstream signaling programs that include increased angiopoietin-2(ANGPT2) expression and dysregulated MAPK pathway activity, with measurable consequences for endothelial permeability and microvascular stability. These observations help reconcile the frequent clinical need for repeated treatment courses and the propensity for partial relapse, and they provide a rationale for linking light-based therapy with strategies that modulate vascular destabilization and treatment-induced revascularization [9, 10].

Based on the molecular pathological mechanisms of PWS, therapeutic strategies are primarily focused on eliminating aberrant vasculature driven by GNAQ mutations. Currently, PDL and PDT are the first-line clinical modalities, both of which rely on physical means to destroy the malformed vasculature. However, PDL and PDT can only target pre-existing vascular structures and are incapable of correcting the sustained activation of MAPK or PI3K signaling pathways or the elevated expression of pro-angiogenic factors such as VEGF, all of which result from GNAQ mutations. Long-term clinical follow-up has revealed that recurrence remains possible even after PDT treatment. Han et al. were the first to report a case of PWS recurrence 22 years following PDT, and through histological analysis, they explored the potential mechanisms underlying recurrence, highlighting the need for clinical attention to long-term relapse risk [11].

PDL is currently the gold standard for the treatment of PWS, with the 595 nm wavelength being the most commonly used in clinical practice [12]. Regarding treatment intervals, favorable outcomes can be achieved within a range of 4 to 10 weeks, with 8 weeks yielding the optimal results. However, when the interval exceeds 10 weeks, the therapeutic efficacy decreases significantly [13]. Early initiation of PDL treatment in infancy is both safe and effective, and may reduce the need for general anesthesia while maximizing the likelihood of achieving clearance before school age [14]. Nevertheless, contemporary analyses and network meta-analytic synthesis indicate that durable and near-complete clearance remains difficult for a substantial proportion of patients, and aggregate outcomes have not improved substantially over several decades, underscoring a persistent efficacy ceiling for conventional photothermolysis alone [15, 16].

Response variability to PDL is clinically significant and multifactorial. In particular, post-treatment angiogenic rebound has been identified as a critical contributor to refractoriness and relapse [17]. While complication rates are generally low, blistering, dyspigmentation, and scarring remain clinically relevant in the context of repeated-session paradigms and cumulative exposure, particularly in darker skin types and in hypertrophic lesions [18]. These limitations motivate interest in combination strategies, objective treatment selection, and mechanism-guided optimization rather than escalation by trial and error. PDT with hemoporfin has gained prominence in parts of Asia as an alternative or complementary modality, with randomized trials and large observational studies reporting substantial therapeutic efficacy in selected phenotypes [19, 20]. However, systematic evidence synthesis and expert commentary underscore ongoing uncertainty regarding optimal dosing, durability, and patient selection, and current evidence does not support indiscriminate prioritization for young children without careful individualized risk–benefit assessment [21].

A central barrier to comparative effectiveness research and precision care is the lack of harmonized outcome measures. Prospective studies have used heterogeneous clinician-rated scales, variable photographic methods, and inconsistent definitions of response, limiting cross-study comparability and weakening inference about durability and relapse [33]. Quantitative imaging tools are therefore increasingly used to characterize vessel depth and caliber and to support parameter selection, including D-OCT for vascular feature quantification and treatment guidance [22–24]. Parallel advances in three-dimensional facial scanning, lesion segmentation, and machine learning create opportunities to integrate surface geometry with colorimetry and vascular imaging to provide reproducible longitudinal endpoints and predictive models of treatment response [25–28]. Collectively, these developments motivate a theragnostic direction in which molecular drivers, translational models, and quantitative clinical readouts are combined to individualize modality selection, dosing, and follow-up, while also generating testable hypotheses about why some lesions remain refractory despite technically adequate treatment delivery.

In this review, we synthesize molecular drivers of PWS pathobiology, summarize clinical evidence across established and emerging interventions, and outline translational models and theragnostic endpoints that can support target validation and precision clinical studies. We also propose a practical workflow for baseline risk stratification, objective response quantification, and pathway-informed iteration of therapy aimed at improving durable outcomes.

## Methods

### Scope and approach

This review integrates mechanistic and translational studies with clinical evidence. Evidence was prioritized by design hierarchy (randomized trials, comparative cohorts, large observational series, and evidence syntheses) and by relevance to treatment selection, endpoint definition, and safety.

### Data sources

We used PubMed-indexed literature and reference chaining to capture seminal discoveries on somatic mosaicism, endothelial signaling, and clinical interventions, supplemented by key consensus statements and network meta-analyses when available.

### Evidence appraisal

Because the primary objective was translational synthesis and theragnostic framing rather than pooled effect estimation, formal risk-of-bias scoring was not applied to all included studies; where available, we prioritized systematic reviews that applied contemporary trial risk-of-bias frameworks and transparent certainty judgments when discussing comparative effectiveness.

### Search strategy

We performed a focused search of PubMed and major guideline repositories through February, 2026, using combinations of the terms “PWS” OR “port-wine birthmark” with “GNAQ”, “ANGPT2”, “MAPK”, “PDL”, “PDT”, “hemoporfin”, “sirolimus”, “optical coherence tomography”, and “Sturge-Weber”. Reference lists of high-yield reviews, consensus statements, and pivotal trials were hand-searched to capture earlier foundational work and to ensure completeness across mechanistic, translational, and clinical domains.

### Study selection and data extraction

Eligible clinical studies included randomized trials, comparative cohorts, and large observational series reporting objective or semi-objective measures of PWS clearance, durability, and safety. Preclinical studies were included when they used lesion-relevant endothelial perturbations or models enabling pathway interrogation. For each included study, we extracted sample size, age group, lesion location and subtype where available, treatment parameters, follow-up duration, outcome definitions, and key adverse events.

### Risk of bias considerations

When comparative inferences were required, we interpreted randomized trials through the lens of the revised Cochrane risk of bias tool for randomized trials and nonrandomized comparative evidence through the Risk Of Bias In Non-randomized Studies of Interventions framework. Methodological quality of evidence syntheses was appraised using the AMSTAR 2 instrument. These frameworks were used to guide narrative weighting rather than to generate pooled summary scores.

## Molecular drivers and microvascular pathobiology

Somatic mosaicism is central to PWS pathogenesis. Landmark genomic analyses identified activating variants in GNAQ within affected tissue, establishing PWS and SWS as mosaic disorders rather than inherited conditions [7]. Mechanistically, endothelial GNAQ activation perturbs downstream signaling and vascular homeostasis, promoting vessel dilation, altered permeability, and maladaptive remodeling. In particular, GNAQ p.R183Q has been linked to increased ANGPT2 signaling and enlarged vessel phenotypes, supporting an endothelial-autonomous driver model [9].

Recent work further implicates MAPK pathway activity and ANGPT2-mediated endothelial barrier disruption in capillary malformations, providing a mechanistic bridge to rational combination therapies that pair vessel-selective ablation with antiangiogenic or pathway-targeted agents to reduce post-treatment revascularization [10]. Cellular localization studies indicate that the canonical GNAQ p.R183Q variant is enriched within the lesional vascular compartment, supporting endothelial cells as the primary mutant cell population and reinforcing the concept of cell-autonomous signaling as an initiating event [6]. In parallel, secondary changes in perivascular cells and extracellular matrix accumulate over time, which may explain the clinical transition from flat macules to hypertrophic and nodular lesions that respond less predictably to vessel-selective photothermolysis [29].

Beyond angiogenic signaling, emerging data suggest that vascular inflammation and barrier dysfunction represent convergent downstream phenotypes of GNAQ-driven activation. MAPK-mediated endothelial permeability, leukocyte recruitment, and proangiogenic cytokine production provide plausible mechanisms for post-treatment revascularization and color relapse [10]. These observations provide a mechanistic rationale for therapeutic strategies that temporally couple physical lesion ablation with short-course signaling pathway modulation during the post-treatment wound-healing window.

## Clinical evidence and treatment landscape

### Risk stratification and baseline evaluation

Consensus statements emphasize early phenotypic triage for SWS risk (forehead and upper eyelid involvement), ophthalmologic surveillance for glaucoma, and multidisciplinary longitudinal care for syndromic cases [30]. Quantitative assessment tools remain heterogeneous across studies, limiting cross-trial comparison; consensus-based core outcome development and objective vascular imaging are therefore high priorities for the field.

Facial distribution should be documented with standardized photography and anatomical mapping, because the anatomic pattern of involvement correlates more closely with embryologic mosaic territories than with traditional trigeminal dermatomal boundaries. Prospective data suggest that hemifacial and median forehead patterns carry higher odds of neuroimaging evidence for SWS than nonmedian linear distributions [31]. Baseline evaluation ideally integrates objective vascular phenotyping, including vessel depth and diameter estimates when available, because these parameters influence light-tissue interactions and can inform wavelength selection, pulse duration, and expectations for clearance. D-OCT studies demonstrate that vessel characteristics differ by clinical color subtype and are measurable noninvasively, supporting a move toward imaging-informed stratification in both trials and practice [22].

### PDL: standard of care, but variable response

PDL is the most established first-line modality and is frequently initiated in infancy to leverage thinner dermis and smaller vessel caliber, with reports of high rates of substantial clearance without general anesthesia in specialized settings [14]. However, longitudinal analyses suggest that, at the population level, treatment outcomes have not improved substantially over recent decades, underscoring persistent challenges in lesion heterogeneity, depth, and post-treatment reperfusion [15].

Determinants of response include lesion location, baseline color, vessel depth, and skin phototype [32]. optical coherence tomography (OCT) imaging has revealed significant heterogeneity in vessel diameter (20–160 μm) and depth across PWB subtypes, supporting individualized parameter selection. These findings highlight the potential of OCT-based vessel profiling to guide laser optimization [22, 33]. From a mechanistic standpoint, incomplete clearance is increasingly attributed to a combination of insufficient energy delivery to deeper ectatic vessels and biologic reperfusion through angiogenic repair. This dual explanation supports a precision framework that integrates optimized energy-based treatment protocols with adjunctive short-course pharmacologic agents to modulate angiogenesis, inflammation, or endothelial survival pathways during the post-treatment remodeling phase. Technical innovations, such as larger spot sizes, may reduce treatment sessions needed for meaningful improvement. In a comparative study, a novel large-spot PDL achieved 50% clearance in 4.3 sessions versus 8.8 sessions with an older device, independent of age or lesion location [34].

### Adjunctive and combination strategies

Topical sirolimus (rapamycin) has been explored to mitigate post-laser revascularization. Sirolimus, a macrolide compound and mTOR pathway inhibitor, effectively suppresses endothelial cell proliferation and inhibits pathological angiogenesis. Theoretically, it may counteract post-PDL reparative vascular remodeling, and has therefore been considered a potential adjunctive agent for combination with PDL [35].

Current clinical evidence on PDL combined with sirolimus for the treatment of PWS is primarily derived from small-sample studies. Artzi et al. employed the Tixel drug delivery system to combine PDL with rapamycin (sirolimus) for PWS treatment, and reported that the combination therapy significantly improved lesion clearance and reduced the number of required PDL sessions [36]. These findings suggest that adjunctive antiangiogenic therapy may enhance the efficacy of PDL in treating PWS by inhibiting post-laser angiogenesis and revascularization. However, not all studies have demonstrated additional benefit. In a prospective intra-patient randomized trial, Greveling et al. found that adding topical rapamycin to PDL did not improve colorimetric clearance at 6 months, with PDL alone achieving the highest clearance among all treatment arms [37]. Of note, Hobayan et al. reported three cases of delayed ulceration and systemic drug absorption following PDL combined with topical sirolimus, indicating potential safety concerns with this combination regimen. Clinical application should therefore balance efficacy and safety, with individualized intervention strategies [35].

Beyond sirolimus, combination approaches have included sequential or dual-wavelength strategies to address deeper vessels, as well as immune and vascular modulators intended to amplify or stabilize laser response [38]. Network meta-analyses comparing light-based modalities underscore substantial between-study heterogeneity in protocols and outcomes, but they also support the concept that no single wavelength is universally optimal across phenotypes [16, 18].

### PDT with hemoporfin (HMME)

emoporfin-mediated photodynamic therapy (PDT) represents an alternative vascular-selective treatment modality, with demonstrated efficacy in randomized controlled trials and large real-world cohorts encompassing both adult and pediatric populations.A phase IIb multicenter trial supported dose selection and identified meaningful clinical responses across dosing arms, while emphasizing the importance of adverse-event monitoring and standardized response metrics [39].

In clinical practice, HMME-PDT is most frequently delivered with 532-nm illumination after systemic photosensitizer administration, targeting vascular endothelium through photochemical injury rather than purely thermal damage. Reported benefits include meaningful blanching in some PDL-resistant lesions, but treatment can be painful and is commonly associated with transient edema, purpura, and post-inflammatory dyspigmentation. Evidence syntheses highlight that comparative certainty remains limited by variable outcome definitions and risk of bias across trials, reinforcing the need for harmonized endpoints and head-to-head designs against optimized PDL protocols [40]. Comparative evidence syntheses suggest that hemoporfin-PDT can achieve higher clearance than PDL in selected phenotypes but may carry higher risks of short-term adverse events, reinforcing the need for individualized risk–benefit assessment [20].

### Emerging targeted agents and pathway-informed concepts

Mechanistic discoveries support exploration of pathway-targeted adjuncts, including MAPK and ANGPT2-axis modulation, to reduce endothelial hyperpermeability and post-ablation reperfusion. Translational work should prioritize skin-delivery feasibility, safety, and combinability with established light-based modalities. A pragmatic short-term goal is to identify druggable nodes that are both proximal to GNAQ signaling and amenable to topical or intralesional delivery. Candidates include MEK inhibition to attenuate ERK-driven permeability, as well as strategies that suppress ANGPT2-mediated vessel destabilization to favor durable remodeling after energy-based vessel injury [10]. Because PWS is a mosaic condition with primarily cutaneous involvement, local delivery paradigms that minimize systemic exposure are particularly attractive, but they require rigorous pharmacokinetic and dermal penetration characterization.

In this context, lesion-stage biology should inform target selection. Early, flat lesions are likely dominated by endothelial signaling dysregulation and microvascular ectasia, whereas hypertrophic or nodular lesions exhibit fibrotic remodeling, increased dermal thickness, and deeper ectatic channels, which may shift the therapeutic emphasis toward strategies that combine deeper vascular targeting with antifibrotic or matrix-modulating approaches.

## Translational models for mechanistic dissection and target validation

Patient-derived endothelial cultures enriched for lesion-specific somatic variants enable direct testing of endothelial-autonomous mechanisms, pathway dependency, and pharmacologic modulation under controlled conditions. In vivo and ex vivo models remain essential to capture vessel maturation, perivascular cell interactions, and hemodynamic forces that shape therapeutic response. Priorities include mosaic and inducible models that recapitulate spatially restricted endothelial activation, as well as engineered microvascular platforms integrating flow and oxygenation to evaluate combination regimens and reperfusion biology.

Genetically engineered approaches that restrict mutant GNAQ expression to the endothelium have been instrumental for establishing causality and for revealing downstream mediators such as ANGPT2.These models enable testing of timing, dosing, and sequencing questions that are difficult to answer in humans, including whether transient pathway suppression around laser sessions can shift the repair program away from reperfusion and toward stable involution.

Engineered human microvascular platforms offer complementary advantages, allowing co-culture of endothelial cells with pericytes and fibroblasts under defined flow and oxygen conditions, with readouts that include barrier integrity, lumen caliber, and dynamic perfusion recovery after photothermal or photochemical perturbation. Such systems are well-suited to screening combination regimens and to identifying biomarkers that translate to noninvasive theragnostic monitoring in patients.

## Theragnostic directions and quantitative endpoints

Theragnostics—integrating diagnostics with therapeutic decision-making—are particularly relevant for PWS because vessel depth, diameter, and flow are key determinants of optical energy deposition and clinical response. D-OCT provides noninvasive quantification of vessel depth and diameter across large cohorts and has identified measurable vascular features that correlate with baseline phenotype and can guide parameter selection.

D-OCT analysis of PWS patients has revealed significant regional differences in vascular characteristics: the superficial vascular plexus is significantly shallower in the head than in the extremities, while vessel diameter and density in the head are higher than those in the trunk and upper extremities [41]. Studies have confirmed substantial intralesional and interlesional vascular heterogeneity in PWS, with mean vessel density and diameter being higher in affected skin than in control skin; the depth range where vessel density consistently exceeded that of control skin was localized to the superficial dermis [42]. For trial readiness, the field needs harmonized endpoints that combine patient-reported outcomes, standardized photographic scoring, and objective vascular imaging or colorimetric measures to reduce bias and enable cross-study meta-analytic comparisons. Laser speckle contrast imaging represents a complementary approach for microvascular perfusion assessment. Recent technical advances have improved depth-resolved imaging capabilities and reduced noise, enabling better visualization of deeper vessels [Morales-Vargas et al.; Hussein & Moazeni]. These developments may facilitate future integration with structural imaging modalities such as D-OCT to support comprehensive treatment monitoring [24, 43].

As the development of clinical prediction models and AI-enabled tools accelerates, adherence to rigorous reporting standards is essential to mitigate optimistic bias and enhance generalizability. The development and validation of such models should follow the TRIPOD + AI statement for transparent reporting. Concurrently, clinical trials evaluating AI-assisted decision support or imaging pipelines must align their protocols and reports with the SPIRIT-AI and CONSORT-AI extensions. At a minimum, future studies should predefine and clearly report their model development protocols, including data handling and intended validation strategies, and should present both discrimination and calibration metrics, along with measures of clinical utility, rather than relying on performance metrics alone.

## Practical theranostic workflow for precision diagnosis and treatment

Based on the distinct vascular pathoanatomy of PWS—including progressive ectasia, variable capillary depth, and phenotypic heterogeneity across age and sites—we propose a practical theragnostic workflow for precision treatment. This algorithm integrates current evidence from laser and PDT, objective vascular imaging where available, and our institutional experience with a large pediatric and adult cohort. The workflow is adaptive, enabling dynamic escalation or de-escalation based on real-time response and tolerability, rather than a fixed regimen. It is applicable across diverse settings, from specialized centers to general clinics, with explicit attention to skin phototype, age-specific tolerability, and long-term surveillance.

- Step 1: Phenotype and risk stratification (distribution, color, thickness/nodularity; SWS risk triage).
- Step 2: Baseline quantitative vascular assessment where available (D-OCT vessel depth/diameter; colorimetry).
- Step 3: First-line modality selection (PDL for superficial erythematous lesions; consider PDT for deeper or violaceous phenotypes where appropriate).
- Step 4: Parameter personalization (spot size, pulse duration, fluence; PDT dose and illumination; analgesia/anesthesia plan).
- Step 5: Early response assessment using objective endpoints (colorimetry, imaging) plus patient-reported outcomes.
- Step 6: Adaptive escalation (device switch, combination strategies, interval adjustments) guided by vascular features and tolerability.
- Step 7: Long-term surveillance for recurrence, hypertrophy, ocular/neurologic complications (when relevant).

Operationally, precision workflows require standardization of photography and colorimetry conditions, explicit documentation of device parameters at each session, and consistent follow-up timing to disentangle immediate post-treatment effects from durable clearance. For infants and young children, early intervention can reduce the need for general anesthesia, but pain control and procedural tolerability must be explicitly planned, particularly for extensive facial lesions.

Higher melanin absorption increases the risk of dyspigmentation and may narrow the therapeutic window. Treatment plans should therefore integrate conservative parameter escalation, rigorous test-spot protocols when uncertainty exists, and close monitoring for pigmentary change. These considerations should also be reflected in trial designs to avoid systematically excluding higher-risk phototypes and to improve generalizability.

In summary, the management of PWS is evolving from a purely light-based approach toward a mechanistically anchored, phenotype-informed, and image-guided precision framework. The integration of molecular insights into GNAQ-driven pathobiology with quantitative vascular imaging and emerging theragnostic tools is reshaping therapeutic decision-making—from baseline risk stratification and parameter selection to response assessment and long-term surveillance. While PDL remains the global standard of care and PDT offers a valuable alternative for selected phenotypes, ongoing challenges including lesion heterogeneity, post-treatment revascularization, and the lack of harmonized outcome measures underscore the need for continued innovation. Advances in patient-derived models, pathway-targeted adjunctive therapies, and machine learning-based prediction tools are poised to address these gaps, moving the field toward more durable, individualized, and mechanism-guided treatment strategies.

To support the clinical and translational evidence synthesis, we summarize key comparative and observational studies in three tables. Table 2 lists randomized and comparative trials that inform first-line and combination treatment selection, with emphasis on study design, intervention, comparator, and principal findings. Table 3 compiles large observational cohorts and evidence syntheses that provide complementary real-world effectiveness and safety data across modalities. Table 4 summarizes emerging theragnostic and digital-endpoint studies, including D-OCT, three-dimensional lesion segmentation, and machine learning-based prediction models, which collectively illustrate the transition toward imaging-informed, phenotype-guided precision management. Together, these tables are intended to serve as a referenced inventory for trial design, modality benchmarking, and theragnostic workflow integration, rather than as a formal quantitative meta-analysis.

**Table 2:** Randomized and comparative studies in PWS therapy

Study	Design/population	Intervention(s)	Comparator	Key findings
Greveling et al. [37]	Prospective intra-patient RCT; 14 patients completed	PDL + topical rapamycin; PDL + Er: YAG + rapamycin; rapamycin alone	PDL alone	No superiority of rapamycin-containing arms over PDL alone for colorimetric clearance at 6 months; feasibility of intra-patient randomized design.
Noormohammadpour et al. [38]	Intra-patient randomized clinical trial; 24 patients	Double-pass PDL (long then short pulse, 20-min interval)	Single-pass PDL	Higher blanching rates and VAS improvement with double-pass protocol; dermoscopy suggested deeper remnant vessels as common residual target.
Sodha et al. [34]	Retrospective comparative cohort; 160 patients (80 per device)	Novel-generation large-spot PDL	Prior-generation PDL	Similar mean improvement but fewer sessions to reach ≥ 50% improvement with large-spot device; device type was key predictor of reaching endpoint.
Zhao et al. [19]	Randomized controlled trial; 440 participants (330 treatment, 110 placebo)	Hemoporfin-PDT	Placebo/control illumination	Hemoporfin-PDT produced significantly higher clinical response than placebo; adverse events were generally transient but required protocolized monitoring.
Wu et al. [39]	Multicenter randomized double-blind phase IIb; 100 participants	Hemoporfin-PDT at 2.5 or 5.0 mg/kg	Dose comparison across arms	Dose-dependent efficacy and safety supported dose optimization; higher dose (5 mg/kg) improved clinical response (75% vs. 40%) with more frequent transient hyperpigmentation (46.0% vs. 14.3%).

**Table 3:** Large observational cohorts and evidence syntheses informing effectiveness and safety

Study	Design/sample	Modality	Endpoints	Key findings
Jeon et al. [14]	Retrospective cohort; 197 infants	PDL initiated in infancy	Clinician-reported clearance; anesthesia avoidance	High rates of substantial clearance in infancy; demonstrates feasibility of office-based infant PDL protocols in specialized centers.
Shi et al. [32]	Retrospective series; 848 Chinese patients (skin types III–IV)	595 nm PDL	Response rates and prognostic factors	Efficacy varied by lesion location and morphology; large cohort underscores phenotype-driven heterogeneity.
Zhang et al. [20]	Multicenter retrospective cohort; 1679 patients	Hemoporfin-PDT	Clinical response and factors	Large real-world dataset supports effectiveness across phenotypes and informs prognostic modeling and patient selection.
Nguyen et al. [16]	Systematic review and network meta-analysis; 27 RCTs, 1033 observations	Laser and light-based therapies	Clearance, patient satisfaction, adverse events	Hemoporfin-PDT ranked highly for clearance but carried higher AE risk; underscores trade-offs across modalities and reporting limitations.
Shi et al. [18]	Systematic review and meta-analysis (to Aug 2022)	PDL safety	Acute and long-term complications	Long-term scarring rates were low; acute reactions common, emphasizing standardized aftercare and complication reporting.

**Table 4:** Theragnostic and digital-endpoint studies (imaging, segmentation, prediction)

Study	Approach	Sample	Endpoints	Key contribution
Mehrabi et al. [22]	Dynamic OCT characterization	108 patients	Vessel depth and diameter	Defined measurable vascular features to stratify lesions and rationalize optical parameter selection.
Waibel et al. [33]	Observational study; 49 scans (PWB and hemangioma), 5 PWB datasets analyzed	OCT imaging of vascular lesions (pre- and post-treatment)	Normal skin for comparison	Key findings: OCT revealed heterogeneous vessel diameters and depths, suggesting fixed pulse durations may be suboptimal; the largest vessels may exceed standard PDL efficacy.
Elsanadi et al. [24]	OCT-measured vessel characteristics	10 participants; 15 lesion areas	Depth/diameter distributions	Provided OCT-derived microvascular metrics across lesion subregions; informs theragnostic feature selection.
Ke et al. [25]	3D scan + deep learning segmentation	3D model patches (non–patient-level)	Segmentation performance; morphology	Developed automated segmentation for facial PWS evaluation; supports standardized digital endpoints.
Wang et al. [26]	Deep learning-enhanced 3D reconstruction (Colmap)	17 patients	3D lesion mapping; segmentation	Enabled comprehensive 3D lesion assessment to support longitudinal tracking and treatment planning.
Yan et al. [27]	Machine learning prediction model (hemoporfin-PDT)	131 patients	Predicted efficacy	Introduced ML-based efficacy prediction to support individualized counseling and regimen selection.

## Data Availability

No datasets were generated or analysed during the current study.
